# Comparative Genomics Unveils Regionalized Evolution of the Faustovirus Genomes

**DOI:** 10.3390/v12050577

**Published:** 2020-05-24

**Authors:** Khalil Geballa-Koukoulas, Hadjer Boudjemaa, Julien Andreani, Bernard La Scola, Guillaume Blanc

**Affiliations:** 1 MEPHI, APHM, IRD 198, Aix Marseille Univ, IHU-Méditerranée Infection, 13005 Marseille, France; khalil.geballa@mio.osupytheas.fr (K.G.-K.); boudjemaa.hadjer@yahoo.com (H.B.); miaguiabidou@gmail.com (J.A.); 2 Aix Marseille Univ., Universite de Toulon, CNRS, IRD, MIO UM 110, 13288 Marseille, France; 3 Department of Biology, Faculty of science of nature and life, Hassiba Ben Bouali University Chlef, 02000 Chlef, Algeria

**Keywords:** Faustovirus, Asfarvirus, nucleo-cytoplasmic large DNA virus, genome evolution

## Abstract

Faustovirus is a recently discovered genus of large DNA virus infecting the amoeba *Vermamoeba vermiformis*, which is phylogenetically related to Asfarviridae. To better understand the diversity and evolution of this viral group, we sequenced six novel Faustovirus strains, mined published metagenomic datasets and performed a comparative genomic analysis. Genomic sequences revealed three consistent phylogenetic groups, within which genetic diversity was moderate. The comparison of the major capsid protein (MCP) genes unveiled between 13 and 18 type-I introns that likely evolved through a still-active birth and death process mediated by intron-encoded homing endonucleases that began before the Faustovirus radiation. Genome-wide alignments indicated that despite genomes retaining high levels of gene collinearity, the central region containing the MCP gene together with the extremities of the chromosomes evolved at a faster rate due to increased indel accumulation and local rearrangements. The fluctuation of the nucleotide composition along the Faustovirus (FV) genomes is mostly imprinted by the consistent nucleotide bias of coding sequences and provided no evidence for a single DNA replication origin like in circular bacterial genomes.

## 1. Introduction

The nucleo-cytoplasmic large DNA viruses (NCLDVs) comprise an expansive and very diverse group of viruses that infect a variety of eukaryotes [1] They are especially notorious because they include the so called giant viruses, with genome sizes exceeding those of many cellular organisms [2]. Most of the NCLDVs replicate in the cytoplasm of infected cells and share several core genes encoding proteins involved in virus morphogenesis and replication. Phylogenetic reconstruction for some these genes supported the hypothesis that the NCLDVs share a common ancestor [3] that could predate the origin of modern eukaryotes [4]. Despite the remarkable progress made in recent years in the methods for isolating, tracking or characterizing new viruses [5,6], our understanding of the diversity and evolution of large and giant viruses is still very fragmentary. Comparative genomics has a leading role to play for exploring the viral world and filling the gaps of our knowledge on these specific issues.

Among NCLDVs, the proposed Faustovirus (FV) genus comprises large DNA viruses isolated using the free-living model amoeba *Vermamoeba vermiformis* (VV) as a host [7]. Their capsids are icosahedral, and their virions are 200–240 nm large [8]. These viruses are related to the African swine fever virus (ASFV), the causative agent of lethal hemorrhagic fever in domestic pigs [9]. In addition, two other Faustovirus relatives have been recently isolated and described. Kaumoebavirus (KV) was also isolated from the VV host but stands phylogenetically outside the ASFV–FV group [10]. Pacmanvirus (PV) has been isolated from *Acanthamoeba castellanii* co-cultures, and its phylogenetic position is nested between ASFVs and FVs [11]. To date, 11 FV strains have been isolated from sewage samples collected in France, Lebanon and Senegal [12]. Their chromosomes have been sequenced, revealing genome sizes ranging between 456 and 491 Kb [7,8,12]. Some FV-like sequences were also identified in metagenomes generated from arthropods as well as from febrile patients, healthy people, and rodents [13]; however, the true nature of the association between FVs and these organisms needs to be clarified in more detail. In addition, two more distantly related NCLDV lineages have recently been isolated from VV host co-cultures, namely Orpheovirus and Tupanvirus [14,15]. The proposed genus names for all mentioned VV viruses and PV await validation by the International Committee on Taxonomy of Viruses.

Faustovirus E12 (F-E12) is the prototype of the genus. Proteomic analyses confirmed the presence of 162 proteins in the mature F-E12 virion [7]. Moreover, cryo-electron microscopy has demonstrated the existence of a double protein layer encapsulating its genome [16]. Using RNA-seq, Cherif Louazani et al. [17] studied gene expression in F-E12 at nine time points over its entire replicative cycle and identified 26 putative splice-site junctions. The combination of genomic, transcriptomic and proteomic data revealed the unique structure of the F-E12 gene encoding the major capsid protein (MCP), one of the most ubiquitously conserved core proteins among NCLDVs [18]. This MCP gene extended over 17 Kb and was riddled with 13 introns [7,16,17], whereas this gene is generally lacking introns in other NCLDVs, excepted in KV [10]. The origin and biological significance of the invasion of introns in the MCP gene of FVs and KV remain unknown.

Here, we report the genome sequences of six novel FV strains and take advantage of the availability of 11 previously sequenced FV strains to conduct a comprehensive comparative genome analysis of this viral group. Using this new data, we clarified the structure and evolution of the MCP gene across FV strains, re-investigated the FV diversity, and uncovered regions of the genomes with contrasting sequence divergence and nucleotide composition. The origin of this specific pattern of genome evolution is discussed.

## 2. Materials and Methods

### 2.1. Sample Collection and Cultures

The six new FV strains reported in this work were isolated from environmental water and sewage samples collected during two sampling campaigns in France and Algeria between 2015 and 2017 (Table 1). After collection, the samples were stored in sterile tubes in a dark room at +4 °C until their inoculation in VV host cultures. Details of the procedure of the isolation and co-culture of the FV strains are described in [7].

### 2.2. Genome Sequencing and Assembly

The viral genomes were sequenced on a MiSeq instrument using a 2 × 251 bp paired-end protocol. The quality-based trimming and removal of contaminant sequences (i.e., Illumina adaptors) in sequence reads was done using the AlienTrimmer program [19]. The cleaned reads were assembled using the SPADES program with default parameters [20]. A few remaining gaps were closed by the sub-assembly of reads recruited beforehand by HISAT alignment [21] on orthologous genomic regions corresponding to the gap and its surroundings (500 bp on both ends) in the most similar FV genome identified by BLASTN.

### 2.3. Genome Analysis

Protein-coding genes were predicted with the GeneMarkS program using the --virus option [22], and potential tRNA genes were searched on the RNAscan-SE On-line server [23]. The structure of the FV MCP gene was determined manually by using the MCP protein sequence of F-E12 (whose coding sequence has been validated by transcriptomics [17]) as a query in TBLASTN searches against the contig sequences. The boundaries between exons and introns were determined based on the returned alignments. GeneMarkS-predicted open reading frames (ORFs) overlapping with the MCP gene exons were removed from the final gene list. We also removed ORFs predicted within MCP gene introns if their length was <100 codons. The same overall procedure was used to resolve the gene structure of the DNA-directed RNA polymerase subunit 1. Protein families were reconstructed using the ORTHOMCL pipeline [24] after identifying homology relationships between FV proteins using BLASTP with the maximal E-value set to 1E-5. Multiple alignments of protein families were generated with MAFFT [25], after which positions containing more than 90% gaps were removed from the alignment. Phylogenetic trees were reconstructed using FastTree [26] with default parameters. We used in-house perl scripts to compute the G-C skew = (G − C)/(G + C) and A-T skew = (A − T)/(A + T) [27] and to draw DNA walks [28]. The principle of DNA walk is to slide along a sequence (x-axis) one nucleotide at a time and to move (i) one pixel up or down on the y-axis if the nucleotide is an A or T, respectively, in the case of A-T walk; (ii) one pixel up or down if the nucleotide is a G or C, respectively, in the case of a G-C walk; or (iii) one pixel up or down if the nucleotide is in a coding sequence on the forward or reverse strand, respectively, in the case of a coding sequence (CDS) walk.

## 3. Results

### 3.1. Context of FV Strain Isolations

A new method for the isolation of VV-infecting viruses has been recently developed based on flow cytometry to detect cytopathogenic effects [29]. We used this approach to identify and characterize new specimens of FVs from environmental samples. In the framework of a pilot sampling campaign of several sites in north-west Algeria and south-east France, six new FV isolates were chosen for genome sequencing. These FV strains were isolated from aquatic environments, including a fresh water sample from a reservoir lake and sewage samples (Table 1).

### 3.2. Genome Structure and Content

Illumina sequencing and sequence assembly produced a single contig for each new FV genome, with sizes ranging from 456.7 Kb (F-VV10) to 479.5 Kb (F-VV63) (Table 1), which is in line with the genome sizes of 11 previously sequenced FVs. Thus, the maximal genome length variation observed within the FV species cluster is 35.2 Kb, which represents 7.4% of the average FV genome size (470 Kb). The GC content of the new genomes (37.7%–39.9%) was also concordant with that of the previously sequenced strains (36.2%–39.8% G + C). The genomic analysis of the very first FV isolates suggested that all of them but F-Liban had a circular chromosome [7,8,12]. Here, we tested the potential circularity of each of the newly sequenced FV genomes by PCR assays using genome-specific primer sets pointing outwards of the contig extremities. None of the PCR assays resulted in amplification. We also carefully examined the positions of paired reads mapped at the extremities of the genome assemblies. We did not find any read pair for which each mate aligned on two distinct ends of the contig. Altogether, these results suggested that the six newly sequenced FVs had linear chromosomes. This observation calls into question the validity of the hypothesis of the circularity of the chromosomes of the first FV isolates. A change in chromosome conformation from circular to linear or vice versa is likely to imply significant adaptations of the underlying replication process. It therefore seems unlikely that FV genomes can frequently switch between these two conformations over relatively short evolutionary periods. We re-examined the chromosome structure of the first FV isolates by looking at their sequencing data. Similar to the newly sequenced strains, none of the first FV isolates had paired reads bridging the two extremities of their chromosome contig. We also designed two new pairs of PCR primers that would normally amplify the region between the extremities of the F-E12 genome if the latter was circular (F-E12 is a prototype FV for which we publish the genome sequence). None of these primer pairs generated a PCR amplification. Thus, based on the reanalysis of their sequences, it appears that our early claim that FVs had a circular chromosome is erroneous and that all sequenced FV genomes to date were, in fact, linear. Furthermore, most FVs have terminal inverted repeats (TIRs) at both ends of their genome. The TIRs have sizes ranging from 61 bp to 687 bp (Table 1) and share high similarity with the genomes of a same clade. TIRs between more distantly related FVs still retain residual nucleotide similarity, suggesting that they all are orthologous. It is unclear whether the lack of TIRs in four FV contigs (F-VV10, F-LC9, F-VV57 and F-VV63) reflects the natural variability of the FV genome structure or a failure of the sequence assembly program to resolve these regions using short reads. The presence of repeated sequences is reminiscent of ASFV [30] and other large DNA viruses such as phycodnaviruses [31] or poxviruses [32], whose genomes are composed of a lineal double-stranded DNA molecule with covalently closed ends and TIRs. Thus, the general organization of the FV chromosomes resembles that of those of other NCLDVs.

**Figure 1:**
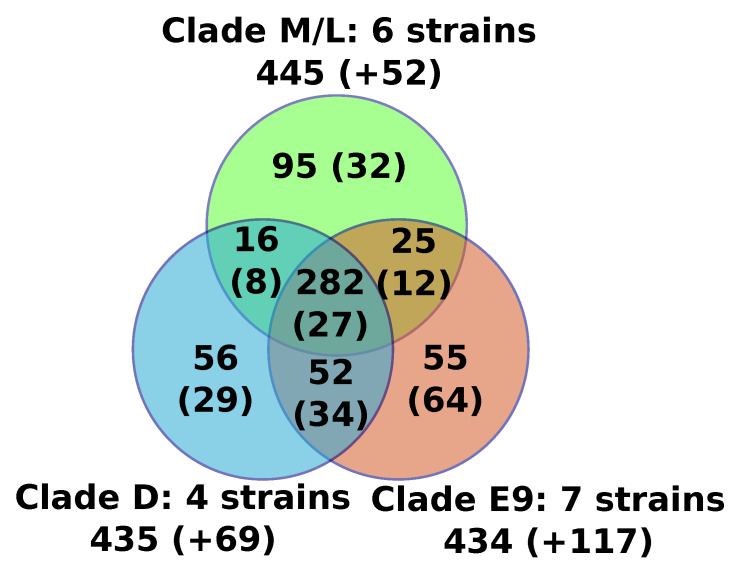
Venn diagram of the numbers of protein families shared between FV clades. For each area of the diagram, the number of protein families ubiquitously conserved in all of the FVs of the corresponding group is indicated. Numbers in brackets indicate additional protein families that were only conserved in a subset of FVs from the relevant group. The names of the FV strains contained in each clade are detailed in Figure 2.

For the sake of consistency, the 17 FV genomes were reannotated using the exact same procedure, and we predicted between 471 (F-VV10) and 506 (F-E9) putative protein genes per genome (Table 1). We found no evidence for the presence of tRNA genes. Furthermore, we found between 12 and 17 introns in the MCP gene (see below), the size of which ranged from 343 to 4635 bp. We also found a single intron of 452 to 507 bp in the DNA-directed RNA polymerase subunit 1 gene of FVs from clades E9 and D. No robust evidence of additional genes containing introns was found specifically in F-E12 by mapping previously generated transcriptome data [17], or in all strains by aligning homologs of FV proteins back to the FV genomes. The predicted proteins of all FVs were assembled into 767 orthologous protein families containing two or more members, including a core set of 282 protein families that were found present in all sequenced FVs (Figure 1, Table S1). An additional set of 20 genome-specific predicted genes were identified (i.e., genes not shared with any other FV), the majority of which encoded short peptides (< 100aa) with no detectable protein similarity in public databases (BLASTP E-value cutoff = 1E-5). The only species-specific gene with a predictable function was F-VV10#87, encoding a probable resolvase most closely related to those found in some mimiviriruses. Another two species-specific genes had uncharacterized functions but shared homology with Catovirus (F-E9#243) and with some eukaryotes (F-D3#210). All together, the six newly sequenced genomes only modestly expanded the FV pangenome with nine gene families and nine genome-specific genes, but only one of the new FV predicted genes (F-VV10#87) had an annotated function. Moreover, based on the genes sharing similarity between viruses of the M/L clade and those of the E9 and/or D clades, we can estimate that the FV last ancestor contained at least 370 genes.

### 3.3. FV Phylogeny and Diversity

**Figure 2:**
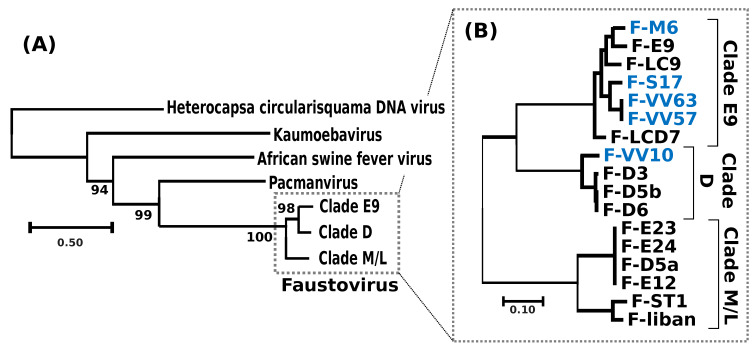
Phylogenetic relationships between FVs and virus relatives. (**A**) Unrooted phylogenetic tree of FVs and virus relatives reconstructed using the viral DNA polymerase as a marker. FV clades have been collapsed for clarity. Details of the clade compositions are given in B. SH-like local supports for branches are indicated beside nodes. The scale bar indicates the number of amino-acid substitutions per site. (**B**) Phylogenetic tree of FVs reconstructed from the concatenation of the alignments of 267 single-copy core FV proteins. All branches received maximal SH-like local support. Names of the newly sequenced FV strains are shown in blue.

A phylogenetic tree reconstructed using the DNA polymerase protein recovered the previously reported phylogenetic position of FVs as a sister group to PV [11] and allowed the identification of the root of the FV clade (Figure 2a). Out of the 282 core proteinfamilies, 267 were encoded by a single-copy gene in every genome and were used to infer the detailed evolutionary relationships between FVs. The resulting phylogenetic tree recovered three main clades, namely E9, D and M/L (Figure 2b); five of the newly sequenced FVs isolated from sewage and lake samples collected in France and Algeria belonged to clade E9, containing other FVs sampled in the south of France; F-VV10, also isolated from Algerian sewage, grouped in clade D, containing other FV strains from Senegal. The overall level of nucleotide similarity between the single-copy core gene coding sequences is high within each clade, typically ranging between 92% and 100% (Figure S1a). The similarity between clades E9 and D is in the order of 70.5% and is 64.5% between E9/D and M/L. The 100% average nucleotide similarity observed between the core genes of the two newly sequenced F-VV57 and F-VV63 strains, both isolated from Algeria, raises the question of a possible contamination by the same virus. However, we found 52 substitutions between the two genome assemblies outside of the core genes plus four large indels totaling 1795 bp (Figure S1b), which ruled out the contamination hypothesis. Two other sets of previously sequenced FVs (i.e., [F-D5b, F-D6] and [F-D5a, F-E12, F-E23, F-E24]) also shared 100% overall similarity between their single-copy core genes. The numbers of genome-wide nucleotide substitutions and the numbers of gapped positions eliminated the hypothesis of contamination for two of them (F-D5b and F-D6: 73 substitutions and 1141 gapped positions). By contrast, the very small number of differences between F-E23 and F-E24 (1 substitution and 0 gapped positions) and between F-E12 and F-D5a (1 substitution and 1 putative insertion of 61 bp at the end of the F-D5a contig) suggested that these strains were either extremely close or identical.

The phylogenetic tree of the 17 sequenced FVs suggests that the diversity of FVs infecting VV is circumscribed to only three apparent clades (i.e., E9, D and M/L). To further explore the viral diversity and the ecological niche of FVs, we aligned the FV MCP, packaging ATPase and DNA polymerase against the IMG/VR database, which compiles viral contigs assembled from >6000 ecologically diverse metagenomic samples [33]. Phylogenetic reconstructions with homologs to the three FV proteins revealed a single contig (id: 3300003402) containing both a packaging ATPase gene and an MCP gene most closely related to clade M/L FVs (Figure S2). This contig was derived from metagenomic samples of wastewater bioreactors used in cyanide and thiocyanate bioremediation [34,35]. Interestingly, the same metagenomic datasets also contained substantial numbers of reads identical to a draft genome sequence of the VV host (Figure S3), suggesting that the association between VV and FVs first revealed in laboratory also exists within the complex microbial communities of artificial environments. Additional homologs of the packaging ATPase were found in metagenomic datasets and grouped at the root of the FV clades—though with relatively low bootstrap support—suggesting that more remote relatives of FVs may exist in environments including black smoker hydrothermal chimneys and municipal landfills. However, the natural hosts of these putative divergent FVs are currently unknown. Thus, the exploration of existing metagenomic datasets failed to reveal a more complex diversity of FVs than that already unveiled by virus isolation and sequencing.

### 3.4. MCP Gene Structure and Evolution

**Figure 3:**
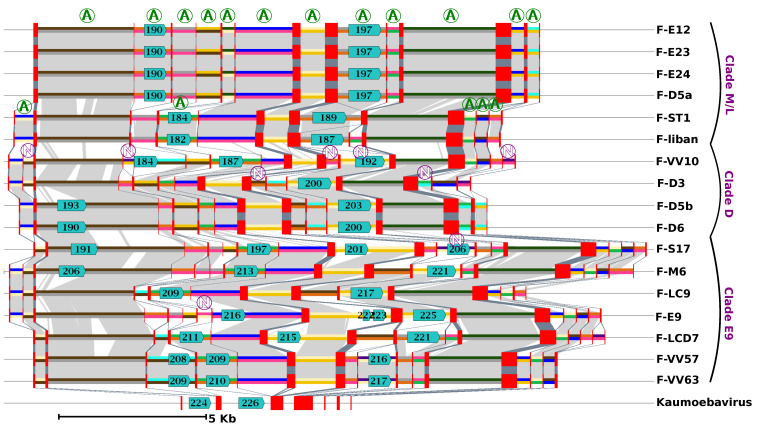
Evolution of the major capsid protein (MCP) gene structure in FVs and Kaumoebavirus (KV). The figure shows a graphical representation of the FV and KV MCP gene alignment. Exons and introns of the MCP genes are shown by red rectangles and 2-colour segments, respectively. Introns represented by the same 2-colour code are orthologous (i.e., share sequence similarity and position relative to the MCP coding sequence). A circled “A” marks orthologous introns that were present in the last FV common ancestor (i.e., introns shared by any virus from clade M/L and any virus from clades D and/or E9). Inversely circled “N”s indicate introns that have no evidence of being present in the last FV common ancestor. Light and dark grey areas indicate significant nucleotide similarity between introns and between exons, respectively. Predicted ORFs with significant protein similarity to group I intron endonucleases are shown with blue arrows with a number inside indicating the ORF id in the respective genome annotation.

A unique feature of FVs compared to other viruses is the large number of introns found in their MCP gene [7,8,17]. Overall, we identified between 13 and 18 introns in this gene across the 17 FV genomes. These numbers contrast with the average NCLDV genes, where the presence of introns is generally sporadic; indeed, when present, introns are generally rather in the order of one to five per gene [36]. We aligned and compared the structures of the FV MCP genes to shed light on the mechanism and dynamics of their evolution. Based on the relative position of introns in the coding sequences, we identified 25 distinct intron insertion sites (Figure 3). None of the intronic flanking sequences follows the GU-AG rule, suggesting that the removal of introns during mRNA maturation is performed by a self-splicing mechanism independent of the host spliceosome. All but two of these intron sites were conserved in two or more viruses, of which 17 were shared between viruses of clade M/L and viruses of clade D or E9, supporting the hypothesis that the last FV common ancestor had at least 17 introns in this gene. However, orthologous introns exhibited various degrees of truncation, sometimes reaching complete deletion, suggesting that intron evolution in the MCP gene followed a birth and death process. Some of these introns, inserted at nine distinct sites (eight of which were inferred to be present in the last FV common ancestor), contained an ORF encoding a homing GIY-YIG endonuclease. No other GIY-YIG endonuclease genes were found elsewhere in the FV genomes; however, two homologs were identified in the first and second introns of the KV MCP gene (YP_009352642 [#224] and YP_009352644 [#226]). None of the KV introns occurred exactly at the same position as the FV introns in the MCP coding sequence, which implies that the homing endonucleases are not orthologous. A homing endonuclease confers mobility on its host intron by binding to and cleaving a defined target site in homologous genes that lack the intron, generating a single- or double-strand break that is repaired using the intron-containing gene as a template [37]. Thus, the invasion of introns into the FV MCP gene may have occurred through recurrent gene conversion events driven by homing endonucleases between MCP alleles having different sets of introns. Multiple alleles of a viral gene may coexist in a host cell when two or more viruses infect the same host at the same time or when a virus infects a host containing MCP gene insertions in its genome. However, although inserted viral genes have been identified in many protist genomes [38], the TBLASTN alignments of the FV endonucleases and MCP against a draft version of the VV host genome did not return a significant match that could support the latter hypothesis.

### 3.5. Contrasting Sequence Divergence along Chromosomes

**Figure 4:**
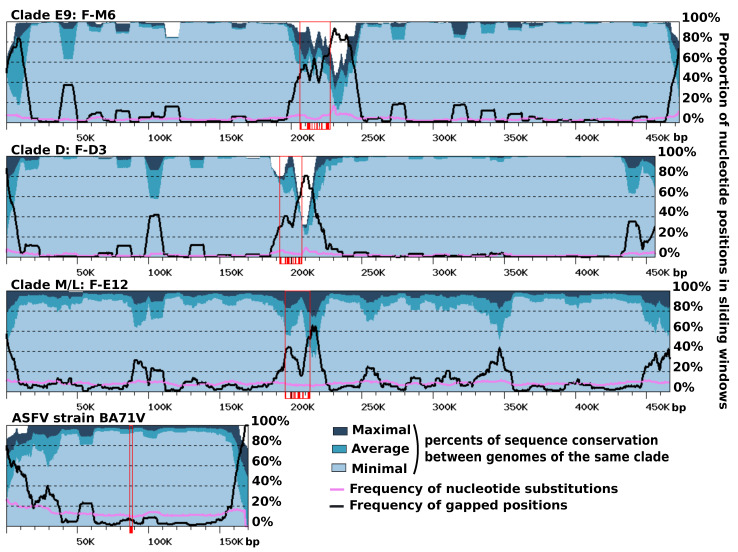
Sequence conservation along viral chromosomes. The graphs represent sequence conservation along reference genomes of FVs (i.e., F-M6 for clade E9, F-D3 for clade D, and F-E12 for clade M/L) and African swine fever viruses (ASFVs) (strain BA71G). Each reference FV genome was aligned against the other FV genomes of the same clade using BLASTN (evalue < 1E-15). The reference ASFV genome was aligned against 12 other sequenced ASFV genomes available in Genbank. The resulting alignments were parsed to compute various statistics within 10 Kb windows slid along the genomes with a 1 Kb step. The dark, medium and light blue areas represent maximal, average and minimal levels of within-clade sequence conservation (global identity) within windows. The mauve and black curves represent the frequencies of positions containing a nucleotide substitution or a gap, respectively, in any alignment within windows. Open red rectangles indicate the positions of the MCP genes, with individual exons shown with shaded boxes below the x-axis.

Gene order was found to be extensively conserved between FV genomes, with only a limited number of genomic rearrangements mostly apparent between FVs belonging to different clades (Figure S4). The ends of contigs contrasted with the rest of the genome. They reflected a more complex evolutionary history, comprising gene inversions and duplications, resulting in locally rearranged gene orders and gene exchanges between the two extremities (Figure S5). Furthermore, the right ends of the F-ST1 and F-Liban contigs contained an almost perfect duplication of an internal genomic segment of 9.5 Kb harboring 10 predicted genes including a putative bifunctional dihydrofolate reductase-thymidylate gene. In addition, we found that discrete regions of reference FV contigs exhibited contrasting levels of sequence divergence in clade-specific similarity plots (Figure 4). This regionalization of sequence divergence was apparent and similarly organized in the genomes of the three FVs clades, suggesting that it was produced by a common mechanism. Typically, the central region of the contigs and their ends showed decreased levels of nucleotide similarity with the other genomes of the same clade, than both the right and left arms of the contig. This trend is particularly visible in the comparisons of clade-E9 genomes and clade-D genomes but slightly less apparent in the comparison of clade-M/L genomes, owing to a lower cumulated divergence between the corresponding viral strains. Further inspection of the nucleotide alignments indicated that the frequency of substitutions is relatively constant along the contigs, including in the central and terminal hypervariable regions. By contrast, the frequency of indels was much more variable and reached maxima that coincide with the hypervariable regions. Thus, the greater sequence divergence observed in the central regions and extremities of the FV contigs appears to result from a greater accumulation of indels rather than a higher rate of nucleotide substitution. The hypervariable central region of the FV contigs carries the MCP gene; thus, one can hypothesize that the higher rate of indels measured in this region might result, in part, from the intron birth and death process evidenced in this gene. By contrast, no evidence of intron accumulation was found in genes located at the contig ends. In this latter region, indels may rather result from an increased genomic rearrangement activity, as evidenced above by the altered order of genes. We finally investigated if other viruses related to FVs exhibit a comparable regionalization of sequence conservation along the genome. The only related viral group for which genomic sequences are available for intra-group comparison and the subsequent construction of a similarity plot is that of the Asfarviridae family containing the African swine fever viruses (ASFV). Pacmanvirus and Kaumoeabavirus are also closely related to FVs, but we could not construct a similarity plot for them because only a single genome sequence is available for these viruses. Using the same procedure as for the FV genome comparison, we observed that the ends of the ASFV chromosome exhibit a higher rate of sequence divergence, here again better explained by a higher rate of indels rather than an increased substitution rate. However, the Asfarvirus genome did not exhibit a hypervariable central region like in the FV genomes, although the MCP gene—which contains no introns in this virus—also occupied a central position in the chromosome. Overall, these results indicate that the chromosomes of FVs and ASFVs show significant differences in their general patterns of evolution.

We next attempted to identify the nature and origin of putative indels by compiling a list of DNA segments from each FV genome for which no significant nucleotide similarity could be found in the other FV genomes (i.e., DNA fragments without matches in a BLASTN search with the E-value cutoff set to 1E-15). In total, we obtained 64 such DNA stretches distributed over the 17 VF chromosomes, with lengths ranging from 50 to 3660 bp and totaling 49.9 kb. These sequences were aligned against the NR database using BLASTX (i.e., DNA translated in the six frames were aligned against a protein database), and 37 of them returned significant matches (E-value < 1E-5). All of these 37 sequences had a match against predicted Fautovirus proteins (excluding self-match), and for all of them but one, a Faustovirus protein was the best match. This indicates that these sequences had homologs in the FV genome(s) but that they were too dissimilar at the nucleotide level to produce a significant match in the initial BLASTN search. This result also suggests that the corresponding indel sequences have been vertically inherited from a FV ancestor rather than acquired by horizontal transfer; however, deletion events among the most closely related FV lineages have made these sequences seemingly unique at the nucleotide level in a single FV genome. Furthermore, a unique DNA sequence from the F-M6 genome (positions 241,080 to 241,592) was most similar to bacterial antitoxin proteins rather than to FV homologs. Unlike the former indels, this sequence might have been acquired through horizontal gene transfer from a bacterium. The remaining 27 DNA stretches that had no significant BLASTX match in NR had a total length of 5.5 Kb and were comparatively shorter (mean length = 200 bp) than those that had a match (mean length = 920 bp); however, their mean GC content (37%) was comparable to that of the whole FV genomes (from 36% to 40%). These sequences were searched against a draft genome of VV that is currently generated in our lab to test the hypothesis of a host origin. Again, none of these sequences returned a significant match using BLASTN. Thus, the origin of these inserts remains unknown.

### 3.6. Strand-Specific Compositional Asymmetries

Variations in nucleotide frequency along a DNA strand reflect the processes that have locally influenced its composition. These processes can be of different natures and affect nucleotide composition through mutational biases or selection pressure. For example, transcription and replication are thought to induce different mutational patterns between each strand [27,39,40]. In addition, the distribution of genes between the two strands plays a role in the formation of nucleotide skews [41]. Compositional bias analysis can reciprocally help in revealing underlying biological processes. For instance, nucleotide skews between complementary nucleotides, (AT skew = (A−T)/ (A + T) and GC skew = (G−C)/(G +C)), are frequently used to determine the position of the origin of replication in bacterial species [27,42,43].

**Figure 5:**
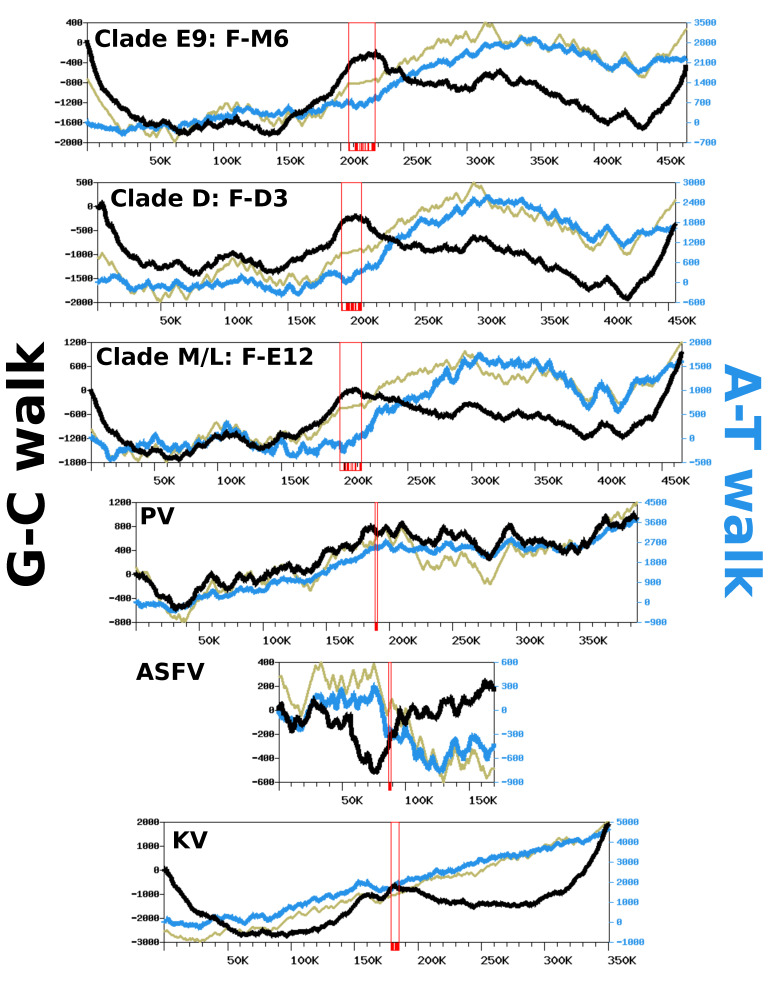
Complimentary nucleotide composition bias along viral genomes. Each graph represents the G-C walk (black), A-T walk (blue) and CDS walk (kaki – y-axis not shown). Open red rectangles indicate the position of the MCP genes, with individual exons shown with shaded boxes below the x-axis. The x-axis units are base pairs. PV: Pacmanvirus A19; ASFV: Asfarvirus BA71V; KV: Kaumoebavirus Sc.

We analyzed nucleotide frequency along the FV genomic sequences using a DNA walk approach [28] to investigate if strand-specific compositional asymmetries exist in this viral group. Figure 5 shows the G-C walk and A-T walk for the reference genomes of each FV clade; the plots for the other FV genomes were perfectly superposable with those of the reference of their clade (not shown), as expected given their high level of nucleotide similarity. Although substantial variation in frequency between complimentary nucleotides is evidenced from the fluctuating shape of the DNA walks, the G-C walk curve did not display the characteristic V-shape obtained for bacterial circular genomes having a single replication origin and bidirectional replication [27]. Rather, the G-C walk curve formed a W-like shape, reflecting three maxima co-localizing with the hyper-variable regions of the genome. This shape indicates that the two ends of the chromosome had opposite G-C biases whereas the MCP gene forms a transition point between two surrounding regions that also have opposite G-C biases. The A-T walk curves exhibited higher amplitudes than the G-C walk curves but without specific structures bounded to the hypervariable regions of the FV genomes.

The same compositional analysis was performed for viruses closely related to FVs, including—by increasing phylogenetic distance—Pacmanvirus (PV), ASFV and Kaumoebavirus (KV). The amplitudes of the respective curves were comparable to those of the FV genomes, except for the KV curves, which were always greater. For both the G-C walk and the A-T walk analysis, the curve shapes were considerably dissimilar between viruses, with the exception of KV for which the G-C walk produced a W-shape similar to the corresponding curves in FVs, with three maxima co-locating on the chromosome extremities and the MCP gene.

To further investigate if the variation in the compositional bias could be an outcome of the replication process, we also computed DNA walks on the intergenic regions alone, which are sequences mostly devoid of transcription and selective constraints. As shown in Figure S6, the G-C and A-T walk curves for the intergenic regions had relatively flat trends. This indicates that replication is unlikely to be involved in the observed compositional bias and that the latter is mostly imprinted within the gene sequences. This hypothesis is further supported by the CDS walk curves (Figure 5), which reflect the distribution of genes between the two DNA strands of genomes. For all viruses, the CDS walk curve seemed to fluctuate in concert with the A-T walk curve, suggesting that the two quantities are correlated. An analysis of the compositional bias in coding sequences confirms this observation. Figure S7 shows that for all viruses, the majority of coding sequences have not only a positive A-T bias but also a positive G-C bias, with the exception of ASFV, which exhibits no specific trend towards a G-C bias in its genes. The same analysis performed on individual codon positions shows that the global compositional bias of coding sequences is a net sum of codon-position-specific biases that have varying intensity and direction. Third codon positions have negative A-T and G-C biases for the majority of genes. By contrast, the first positions of codons have negative A-T and G-C biases, whereas the second positions have a positive A-T bias and a negative G-C bias. Because of the redundancy of the genetic code, the third nucleotide in a codon is the least selectively constrained; this suggests the observed compositional bias at this position could result from mutation biases induced by transcription and/or by a weak selective pressure on synonymous codon usage. By contrast, the nucleotide frequencies at the first and second codon positions, which determine the nature of the encoded amino acid, are under stronger selective constraints. All together, these results indicate that the variation in the nucleotide frequency across these viral genomes is mostly driven by the distribution of protein genes between the two DNA strands, owing to a relatively consistent compositional bias in coding sequences, potentially resulting from a combination of mutational biases and selective pressures.

## 4. Discussion

The sequencing of six new FV genomes and their comparison with those already available allowed us to better delineate the phylogenetic and genetic diversity of this group of large double-stranded DNA viruses. Sequences obtained from various sources, whether by the genomic sequencing of isolated strains or by the metagenome sequencing of more complex microbial communities, suggested that the diversity of FVs is limited to three clades within which genetic diversity is moderate. For this reason, the sequencing of six novel FV genomes did not expand the FV pangenome in a spectacular way (i.e., only nine gene families and nine genome-specific genes). The environments from which FVs have been isolated were always associated with aquatic ecological niches impacted by human activities (sewage, wastewater, artificial lakes, and urban sea shores). These environments were likely to contain VV hosts, reportedly the most common free-living protists found in human environments [44]. In support of this hypothesis, our study indicates that metagenomes containing substantial numbers of FV reads also contained an even higher number of VV reads.

Our study also revealed a regionalized FV genome evolution, in the sense that three distinct regions— i.e., the two chromosome ends and the middle of the chromosome—accumulated indels more frequently than the rest of the genome, while retaining a frequency of nucleotide substitution that was fairly constant. The central hyper-variable region contained the MCP gene, one of the most universally conserved NCLDV core genes. To our knowledge, such an organization of sequence divergence has not yet been described in viral genomes, although other patterns of chromosome evolution have been evidenced in some NCLDV lineages [45,46]. It is remarkable that the FV MCP genes contained between 13 and 18 introns, while viruses are generally devoid of introns in this gene. We showed that the acquisition of most introns was likely mediated by homing endonucleases encoded within some introns and probably predated the last common FV ancestor; nevertheless, comparative genomics also indicated that intron birth and death have occurred since the separation of the FV strains, contributing to the hyper-variability of the central region. Interestingly, the MCP gene of the KV, which also lies centrally in the genome, was found to contain six introns that are not orthologous with those of FVs. It is therefore unlikely that KV and FV introns have been inherited from a common ancestor, but rather, they were independently acquired possibly more recently. Because these two viral lineages infect VV, it is possible that they have acquired their introns in this host, which may serve as a hot spot for intron acquisition. However, three other distantly related viral genera infecting VV hosts, namely Tupanvirus [47], Yasminevirus [48] and Orpheovirus [14], have no introns in their MCP gene. The Yasminevirus genome encodes a homing endonuclease whose gene is located within an intron of the RNA polymerase subunit 1 gene. This intron is at a different position from the intron of the FV RNA polymerase subunit 1 gene, indicating that it is not orthologous to the FV intron.

Variations in the nucleotide composition along FV genomes have not allowed the identification of signals similar to those recorded in bacterial genomes that have a single origin of replication. This suggests that the replication of FV genomes could begin at multiple loci, which prevents the establishment of a nucleotide bias induced by a replication-related constant mutational bias. This hypothesis echoes earlier experimental studies that have shown that the replication initiation of the closely related ASFV can occur in different regions of its genome [49]. Interestingly, FV, PV and ASVF intergenic regions exhibited almost no nucleotide bias, which is an expected outcome under the hypothesis of multiple or random replication origins in these genomes. Our analysis also revealed that one of the major factors contributing to compositional bias along the genomes is the distribution of genes between the two strands, because coding sequences tend to have a global excess of G versus C, and a global excess of A versus T. Furthermore, we showed that this trend is not universally distributed over the three positions of codons, which evolve under different strengths of selective pressure, but is the net sum of codon-position-specific biases that have varying intensity and direction.

## Figures and Tables

**Table 1:** Genomic features of Faustoviruses (FVs).

Strain	Clade	Sampling Site	Genbank ID	Contig Length (bp)	G + C%	Gene no.	Family no.	No. of Introns MCP	TIR (bp)
F-S17	E9	Oran, Algeria, sewage	MN830296	476,423	39.6	486	482	18	249
F-M6	E9	Marseille, France, sewage	MN830295	472,803	39.8	492	485	17	372
F-VV57	E9	Telmcen, Algeria, reservoir lake	MN830297	478,172	39.7	497	491	15	61
F-VV63	E9	Chlef Marsa, Algeria, sewage	MN830298	479,542	39.7	498	490	15	687
F-LCD7	E9	La Ciotat, France, sewage	MN830294	477,407	39.9	502	495	14	247
F-LC9	E9	La Ciotat, France, sewage	CZDJ02000001-5	470,873	39.8	500	492	14	0
F-E9	E9	Marseille, France, sewage	MT335755	491,024	39.6	506	498	16	489
F-VV10	D	Mostaganem, Algeria, sewage	MN956669	456,728	37.7	471	456	17	0
F-D3	D	Dakar, Senegal, sewage	KU556803	455,803	37.8	481	476	16	380
F-D5b	D	Dakar, Senegal, sewage	KU702949	464,523	37.7	488	481	14	324
F-D6	D	Dakar, Senegal, sewage	KU702951	462,011	37.7	485	479	14	309
F-D5a	M/L	Dakar, Senegal, sewage	KU702950	466,051	36.2	474	472	13	528
F-E12	M/L	Marseille, France, sewage	KJ614390	466,265	36.2	474	472	13	498
F-E23	M/L	Marseille, France, sewage	KU702952	465,956	36.2	474	472	14	528
F-E24	M/L	Marseille, France, sewage	KU702948	466,012	36.2	474	472	13	556
F-ST1	M/L	St Pierre de Mezoargues, France, wastewater	LT839607	470,659	36.7	495	467	13	0
F-Liban	M/L	Tripoli El Mina, Lebanon, sea water	MN534311	470,731	36.7	478	465	13	0

## Supplementary Materials

The following are available online at https://www.mdpi.com/1999-4915/12/5/577/s1, Figure S1: Sequence similarity between FV strains, Figure S2: Phylogenetic placement of homologs to FVs in metagenomic datasets, Figure S3: Metagenomic read mapping on the FV and VV genomes, Figure S4: Gene colinearity between FV genomes, Figure S5: Gene colinearity at contig extremities, Figure S6: Complimentary nucleotide composition bias in intergenic regions, Figure S7: A-T and G-C skews in virus coding sequences. Table S1: Orthologous protein families identified with OrthoMCL.
