# Supplementary material for: Comparative Genomics Unveils Regionalized Evolution of the Faustovirus Genomes
Source: Viruses. 2020 May 24;12(5):577. doi: 10.3390/v12050577 (PMC7290515; doi:10.3390/v12050577)
Supplement: Supplementary file 1 [file viruses-12-00577-s001.zip › viruses-785076-supplementary.pdf]

A

Coding sequence

Third codon position

	F-E9	F-LC9	F-M6	F-S17	F-VV63	F-VV57	F-LCD7	F-VV10	F-D3	F-D5b	F-D6	F-E23	F-E24	F-D5a	F-E12	F-ST1	F-Liban
F-E9		98.5	98.8	98.3	98.3	98.3	98.2	70.5	70.6	70.6	70.6	64.2	64.2	64.2	64.2	64.1	64.1
F-LC9	96.8		98.6	98.4	98.3	98.3	98.5	70.6	70.5	70.5	70.5	64.2	64.2	64.2	64.2	64.2	64.1
F-M6	97.4	96.9		98.4	98.3	98.3	98.4	70.6	70.6	70.6	70.6	64.2	64.2	64.2	64.2	64.2	64.1
F-S17	96.5	96.6	96.7		98.9	98.9	98.4	70.6	70.5	70.5	70.5	64.2	64.2	64.2	64.2	64.2	64.1
F-VV63	96.5	96.5	96.6	97.4		100	98.4	70.5	70.5	70.5	70.5	64.2	64.2	64.2	64.2	64.3	64.2
F-VV57	96.5	96.5	96.6	97.4	100		98.4	70.5	70.5	70.5	70.5	64.2	64.2	64.2	64.2	64.2	64.2
F-LCD7	96.4	96.9	96.9	96.8	96.6	96.6		70.5	70.5	70.5	70.5	64.3	64.3	64.3	64.3	64.3	64.2
F-VV10	49.8	49.8	49.8	49.8	49.7	49.8	49.8		98.4	98.5	98.5	64.8	64.8	64.8	64.8	64.7	64.7
F-D3	49.9	49.8	49.9	49.8	49.7	49.8	49.8	97		99.9	99.9	64.9	64.9	64.8	64.8	64.8	64.7
F-D5b	49.9	49.8	49.9	49.8	49.8	49.8	49.8	97	99.9		100	64.9	64.9	64.8	64.8	64.8	64.7
F-D6	49.9	49.8	49.9	49.8	49.8	49.8	49.8	97	99.9	100		64.9	64.9	64.8	64.8	64.8	64.7
F-E23	44.7	44.7	44.7	44.7	44.7	44.7	44.8	45.5	45.5	45.5	45.5		100	100	100	92.2	92.3
F-E24	44.7	44.7	44.7	44.7	44.7	44.7	44.8	45.5	45.5	45.5	45.5	100		100	100	92.2	92.3
F-D5a	44.7	44.7	44.7	44.7	44.7	44.7	44.8	45.5	45.5	45.5	45.5	100	100		100	92.2	92.3
F-E12	44.7	44.7	44.7	44.7	44.7	44.7	44.8	45.5	45.5	45.5	45.5	100	100	100		92.2	92.3
F-ST1	44.5	44.6	44.6	44.6	44.7	44.7	44.6	45.2	45.3	45.3	45.3	82.1	82.1	82.1	82.1		98.9
F-liban	44.5	44.6	44.6	44.6	44.7	44.7	44.6	45.2	45.3	45.3	45.3	82.4	82.4	82.4	82.4	97.5	

B

Genomic nucleotide substitutions

Gapped genomic positions

	F-VV63	F-VV57	F-D5b	F-D6	F-E23	F-E24	F-D5a	F-E12
F-VV63		52						
F-VV57	1745							
F-D5b				73				
F-D6			1141					
F-E23						1	82	82
F-E24					0		82	81
F-D5a					110	109		1
F-E12					157	213	61	

Figure S1: Sequence similarity between FV strains
(A) this table reports the average percent nucleotide similarity between the coding sequences of single-copy FV core genes (upper triangle) or the third codon positions of the single-copy FV core genes (lower triangle). (B) this table reports the genome-wide number of substitutions (upper triangle) and the genome-wide number of gapped positions (lower triangle) between the FV pairs that have 100% nucleotide similarity in (A).

A) MCP

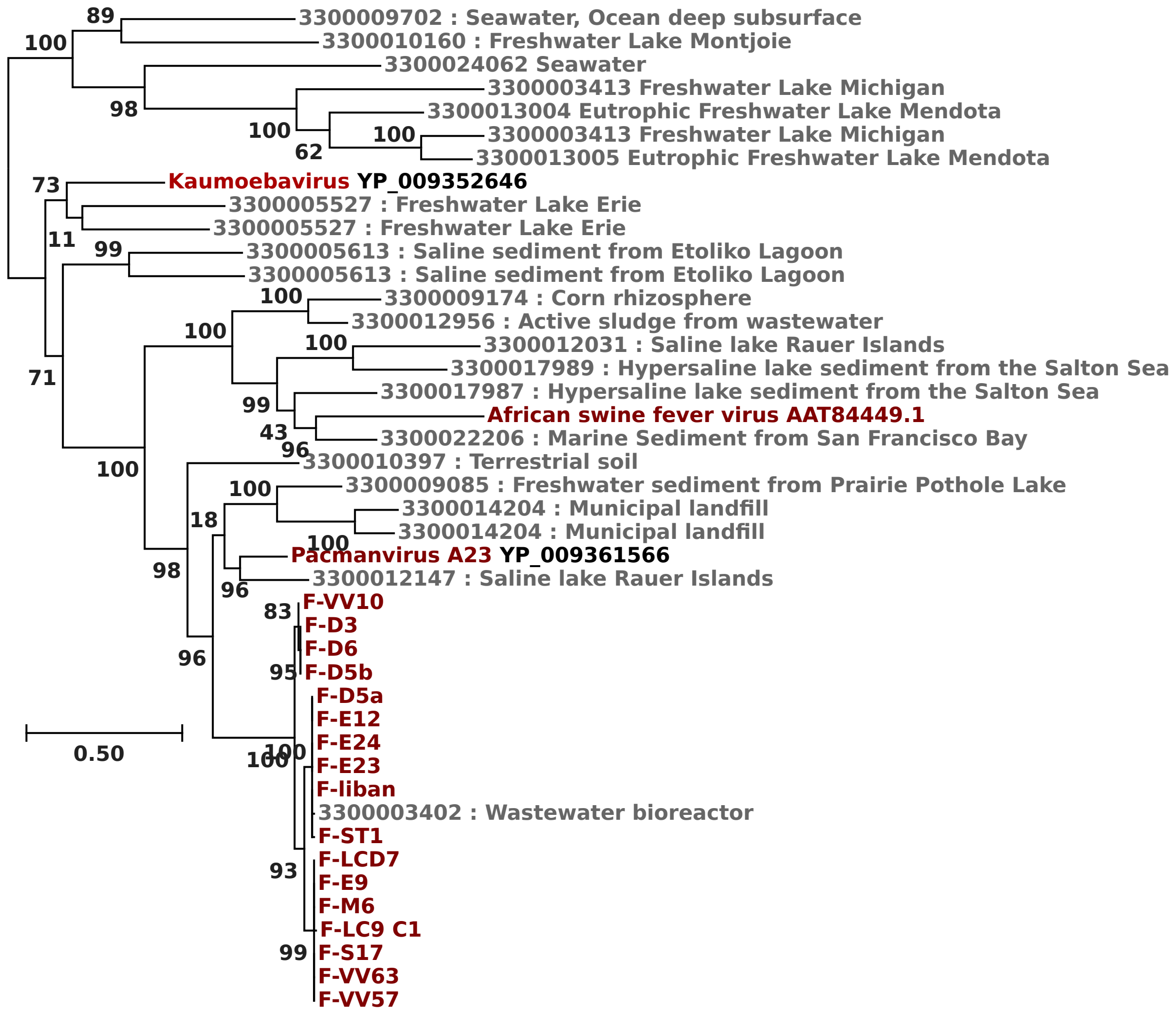

B) ATPase

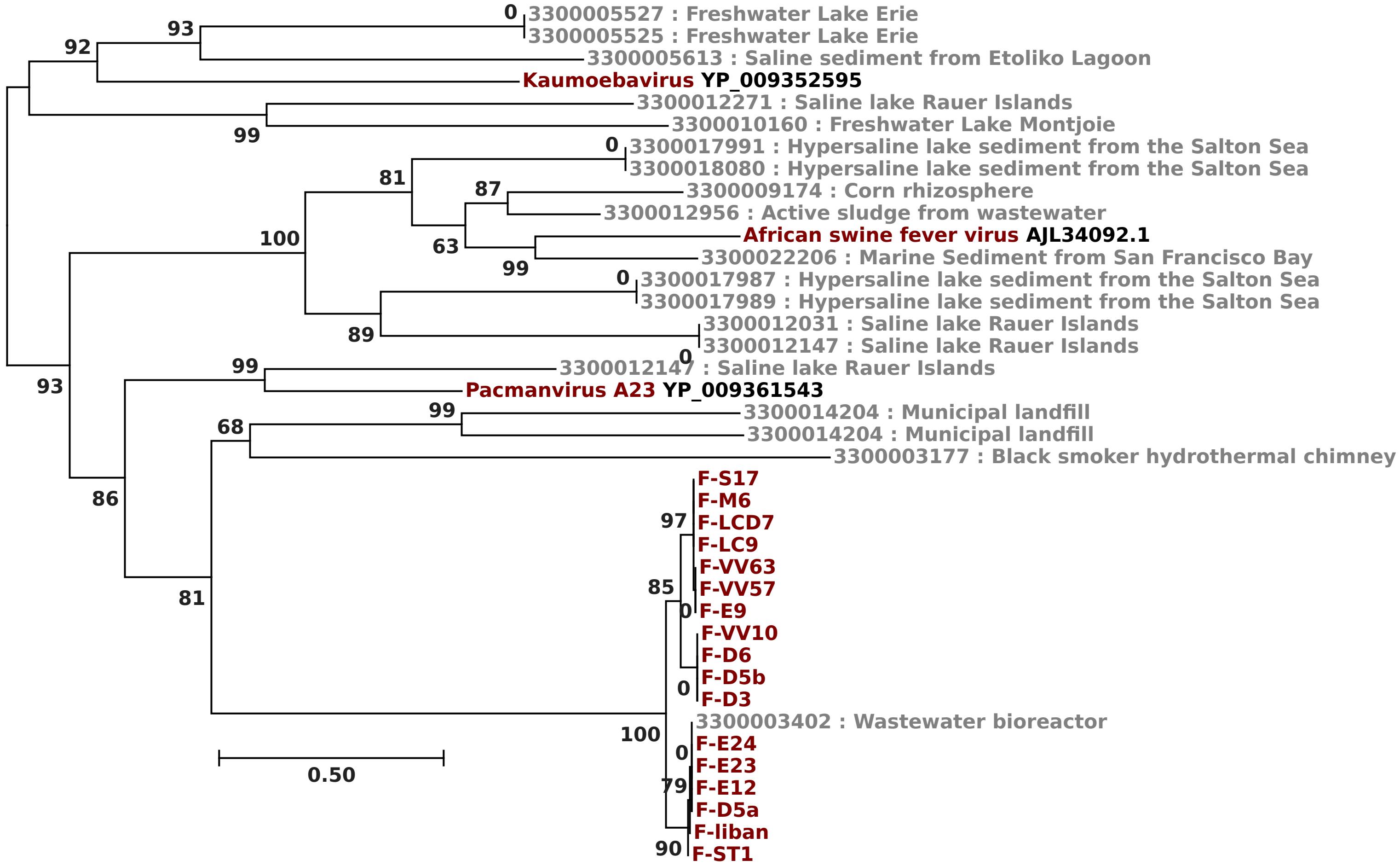

Figure S2, continued

C) DNAP

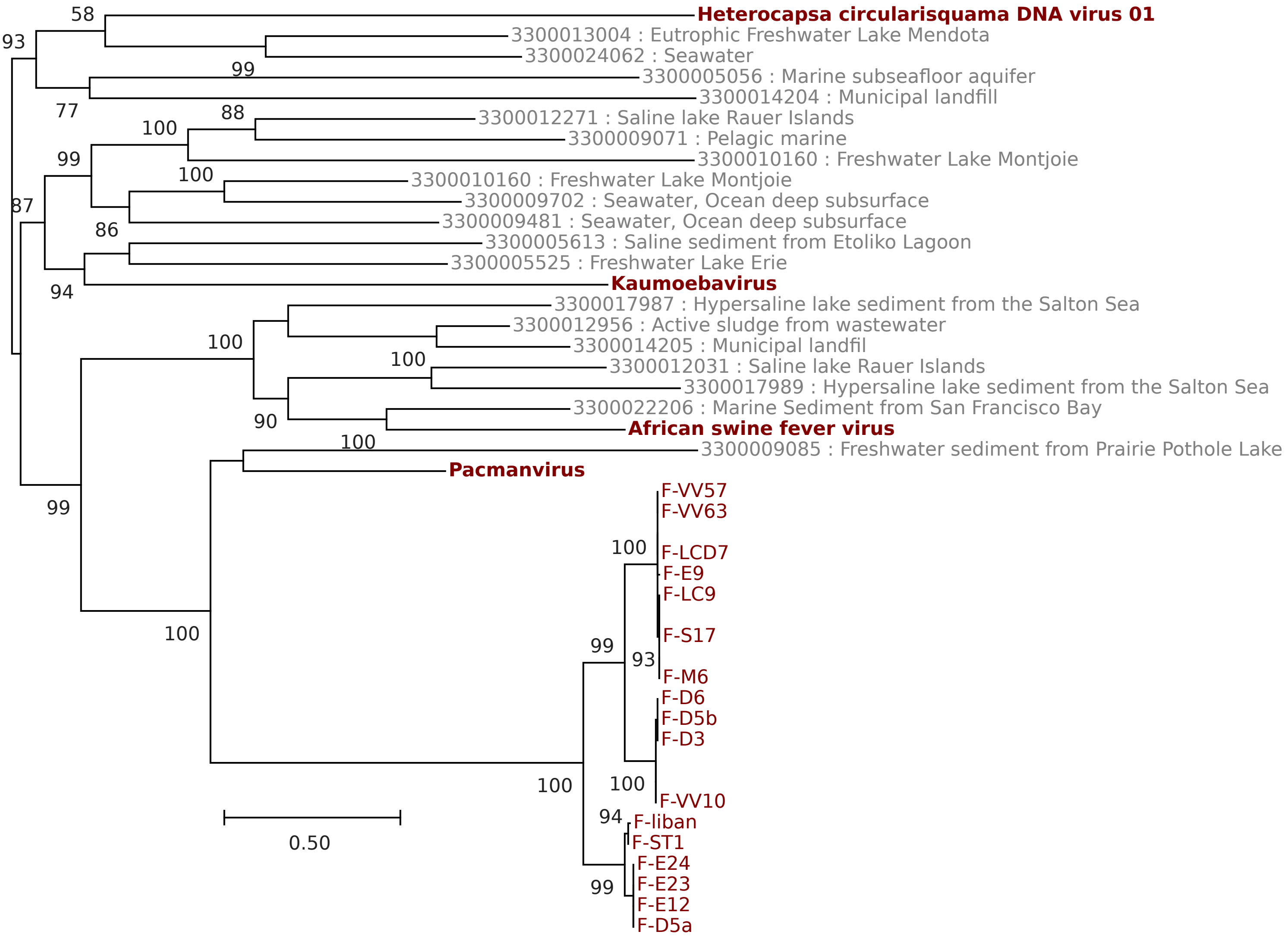

Figure S2: Phylogenetic placement of homologs to FVs in metagenomic datasets The phylogenetic trees of A) MCP, B) packaging ATPase, and C) DNA polymerase are shown with viral sequences indicated in red, and metagenomic sequences in grey. IMG VR database ids are given for metagenomic sequences along with details on the origin of the metagenomic sample. SH-like local supports for branches are indicated beside nodes.

A

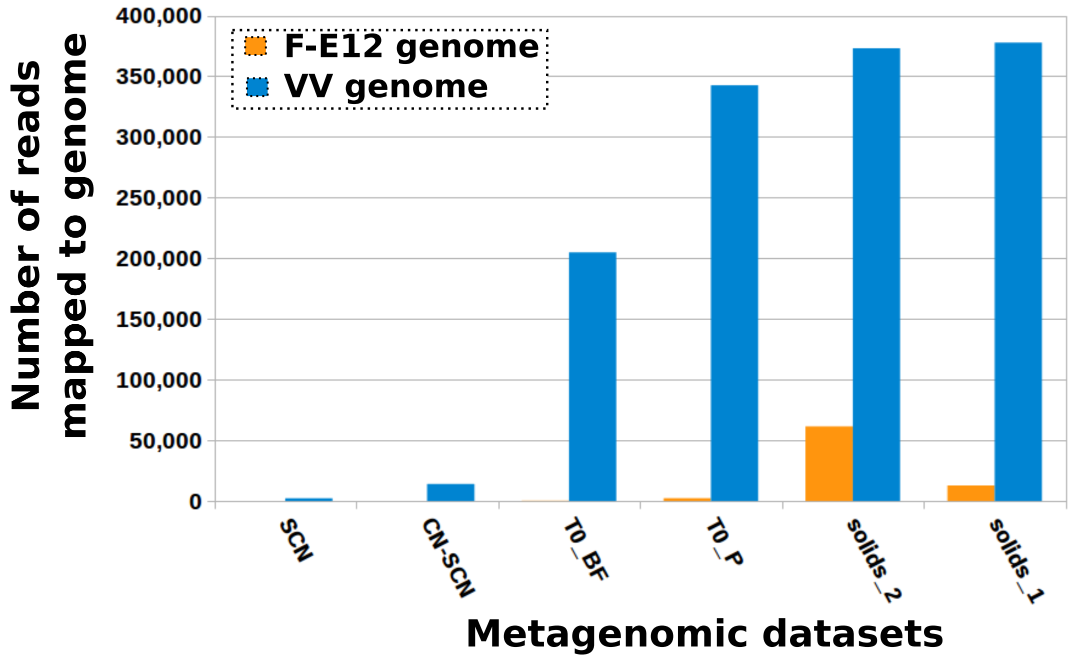

B

Library name	F-E12 reads	VV reads	Sample Ids	Metagenome description
SCN	1	2,264	SRR1955914	Thiocyanate bioreactor
CN-SCN	10	14,030	SRR1955887	Cyanide and thiocyanate bioreactor
T0_BF	250	204,890	SRR3901694	Thiocyanate stock reactor biofilm
T0_P	2,311	342,536	SRR3901695	Thiocyanate stock reactor planktonic
solids_2	61,466	373,009	SRR3997478	Sample from bioreactor
solids_1	12,825	377,667	SRR3997477	Sample from bioreactor

Figure S3: Metagenomic read mapping on the FV and VV genomes
(A) The graph shows the number of metagenomic sequencing reads mapped to the F-E12 genome (red bars) and the VV genome (blue bars). The metagenomic data come from studies of bioreactors containing microbial communities used for the treatment of wastewater (Kantor et al. 2015; Rahman et al. 2017). (B) The Table lists details of the metagenomic datasets, including the numbers of reads mapped to the F-E12 and VV genomes, the SRR genebank id, and the nature of the environment sample.

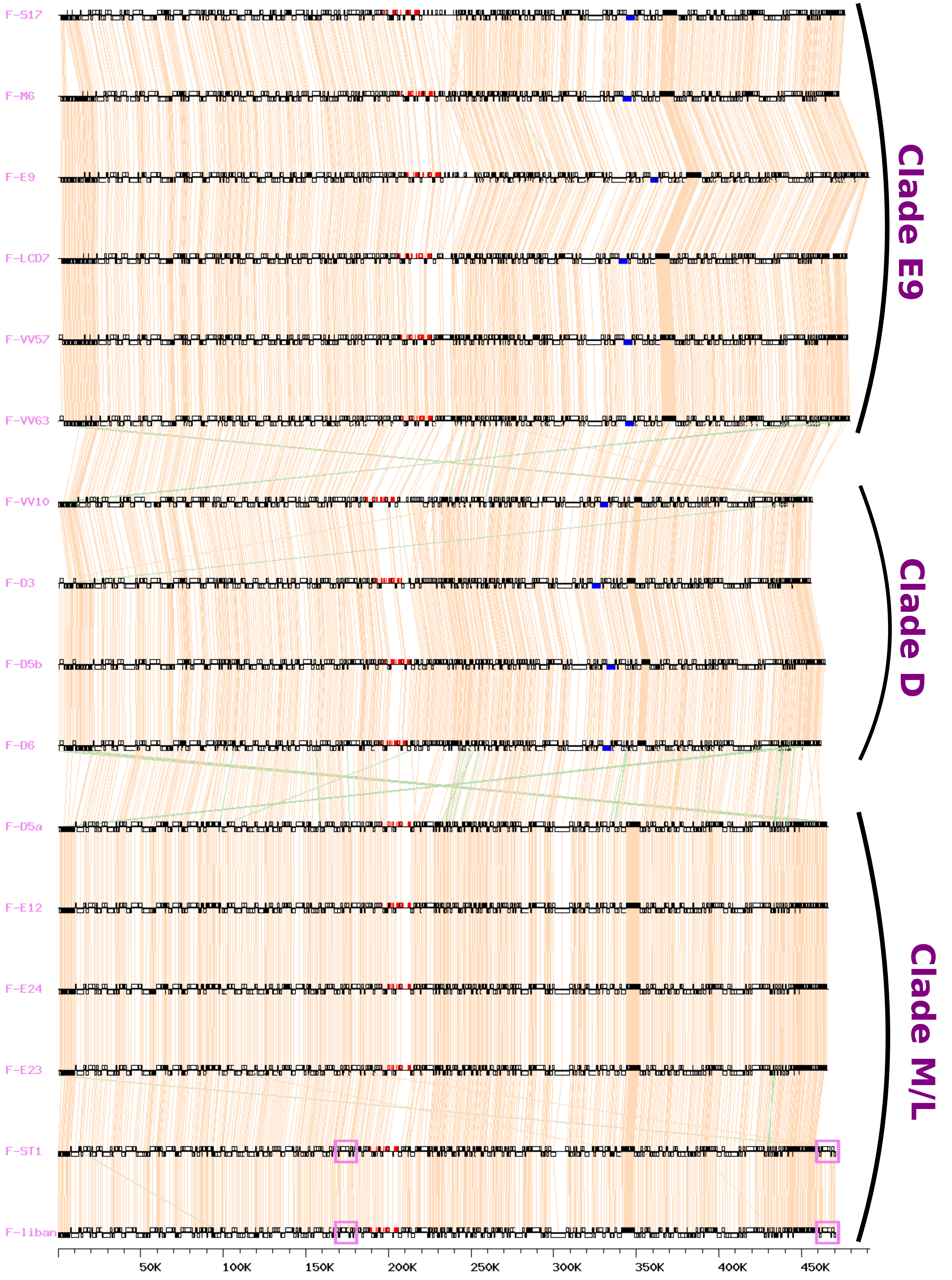

Figure S4: Gene colinearity between FV genomes.
Predicted protein genes are shown with open black boxes. Exons of the MCP and RNA polymerase subunit genes are shown by red and blue filled boxes respectively. Lines join pairs of reciprocal best BLASTP hits between adjacent genomes. Orange lines indicate hits in the same orientation in both genomes; green lines indicate hits in opposite orientation. Purple boxes show duplicated regions in F-liban and F-ST1.

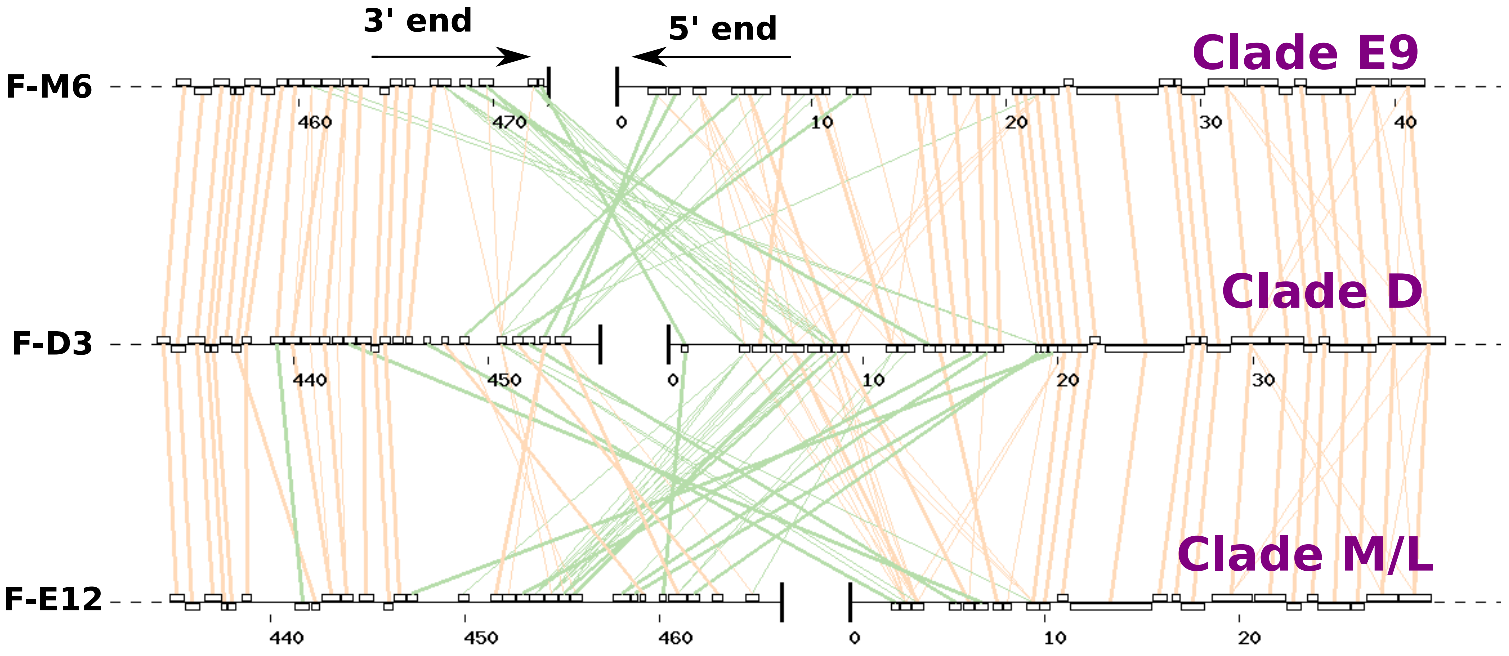

Figure S5: Gene colinearity at contig extremities.

Predicted protein genes are shown with open boxes. Thick lines join pairs of reciprocal best BLASTP hits between adjacent genomes. Thin lines represent additional significant protein similarity between genes (BLASTP evalue 1E-5). Orange lines indicate genes in the same orientation in both genomes; green lines indicate genes in opposite orientation. Numbers below indicate coordinates along the contig.

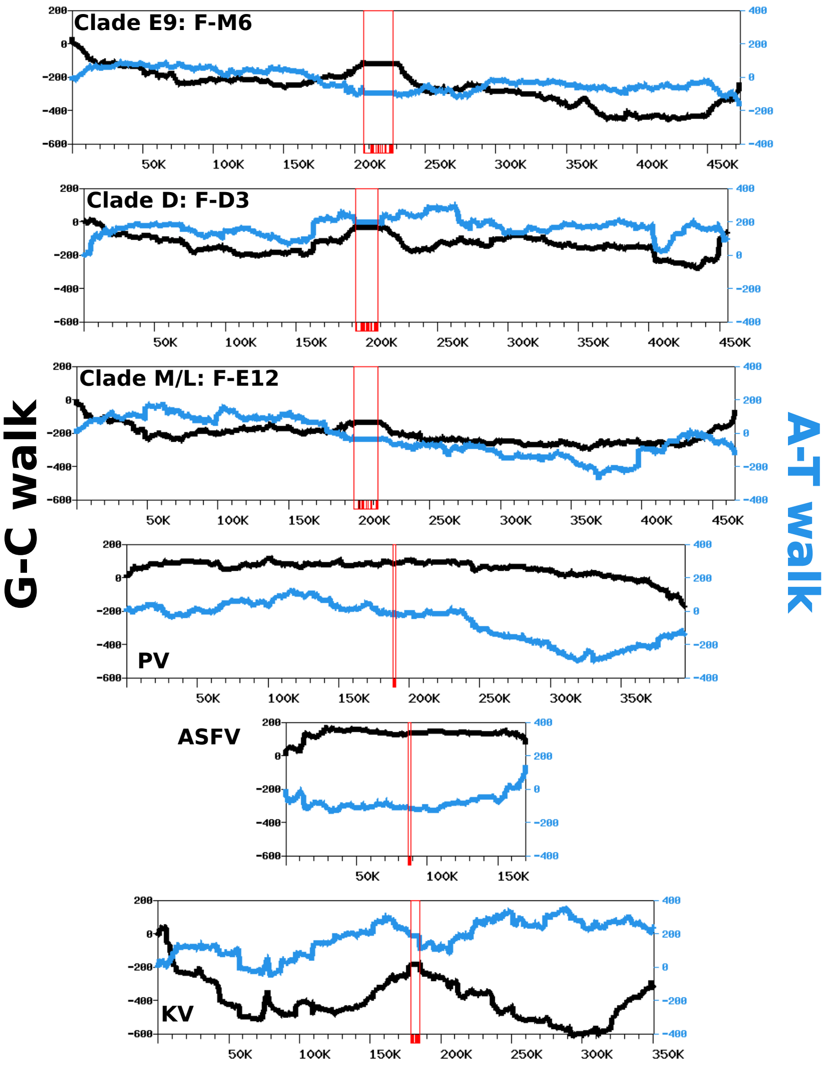

Figure S6: Complimentary nucleotide composition bias in intergenic regions. Each graph represents the G-C walk (black), A-T walk (blue) and CDS walk (kaki – y-axis not shown). Open red rectangles indicate the position of the MCP genes, with individual exons shown with shaded boxes below the x-axis. The x-axis unit is expressed in base pair. PV: Pacmanvirus A19; ASFV: Asfarvirus BA71V; KV: Kaumoebavirus Sc.

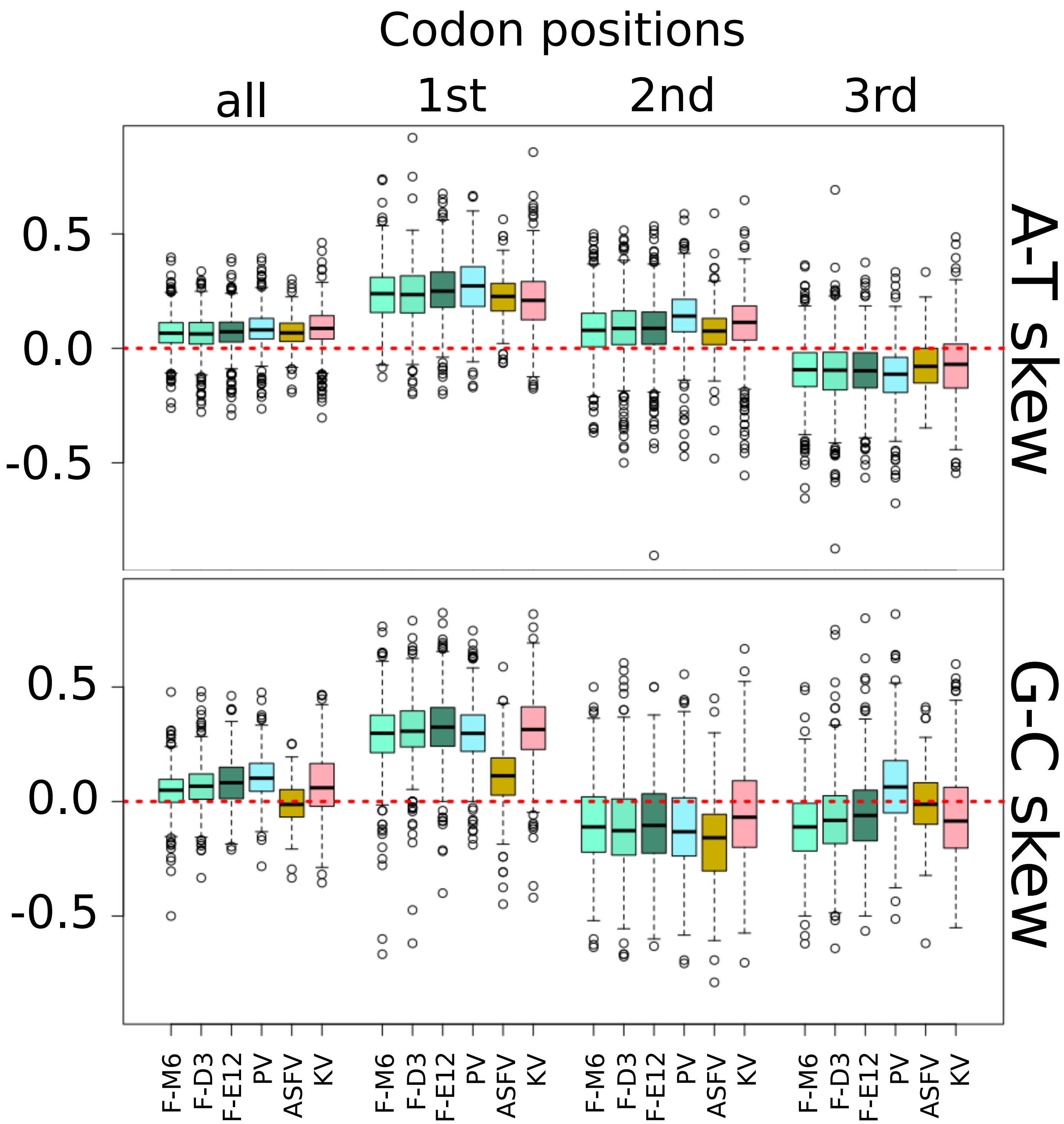

Figure S7: A-T and G-C skews in virus coding sequences. Boxplots represent the distribution of A-T and G-C skews for genes at different codon positions. The horizontal black line within bow indicate the median value. PV: Pacmanvirus A19; ASFV: Asfarvirus BA71V; KV: Kaumoebavirus Sc.
